# Activation of endogenous neural stem cells for multiple sclerosis therapy

**DOI:** 10.3389/fnins.2014.00454

**Published:** 2015-01-20

**Authors:** Iliana Michailidou, Helga E. de Vries, Elly M. Hol, Miriam E. van Strien

**Affiliations:** ^1^Department of Astrocyte Biology and Neurodegeneration, The Netherlands Institute for Neuroscience, An Institute of the Royal Netherlands Academy of SciencesAmsterdam, Netherlands; ^2^Department of Molecular Cell Biology and Immunology, VU University Medical CenterAmsterdam, Netherlands; ^3^Center for Neuroscience, Swammerdam Institute for Life Sciences, University of AmsterdamAmsterdam, Netherlands; ^4^Department of Translational Neuroscience, Brain Center Rudolf Magnus, University Medical Center UtrechtUtrecht, Netherlands

**Keywords:** multiple sclerosis, neurogenesis, gliogenesis, neural stem cells, therapy

## Abstract

Multiple sclerosis (MS) is a chronic inflammatory disorder of the central nervous system, leading to severe neurological deficits. Current MS treatment regimens, consist of immunomodulatory agents aiming to reduce the rate of relapses. However, these agents are usually insufficient to treat chronic neurological disability. A promising perspective for future therapy of MS is the regeneration of lesions with replacement of the damaged oligodendrocytes or neurons. Therapies targeting to the enhancement of endogenous remyelination, aim to promote the activation of either the parenchymal oligodendrocyte progenitor cells or the subventricular zone-derived neural stem cells (NSCs). Less studied but highly potent, is the strategy of neuronal regeneration with endogenous NSCs that although being linked to numerous limitations, is anticipated to ameliorate cognitive disability in MS. Focusing on the forebrain, this review highlights the role of NSCs in the regeneration of MS lesions.

## Introduction

Multiple sclerosis (MS) is a chronic inflammatory, demyelinating disease of the central nervous system (CNS) that has an important neurodegenerative component. The pathological hallmark of MS is the presence of demyelinating lesions (Noseworthy et al., 2000). MS was traditionally considered as a disease affecting only the white matter (WM) areas of the CNS; more recent studies however, showed extensive pathology also in the gray matter (GM) (Geurts and Barkhof, 2008).

MS development is linked to loss of the blood brain barrier (BBB) integrity and migration of autoreactive T-cells and monocytes (Sospedra and Martin, 2005; Hemmer et al., 2006; Vogel et al., 2013). Autoimmunity plays a central role in the disease pathogenesis either as the primary cause or as the consequence of an ongoing neurodegenerative process. Notably, several studies in post-mortem MS brain material and experimental autoimmune encephalomyelitis (EAE) indicated that key features of neurodegeneration, such as neuronal cell atrophy, axonal transection, and neuronal death, already occur in early disease phases (Trapp et al., 1998). The diffuse neuronal damage is associated with pronounced atrophy, decreased functional connectivity and cognitive decline in the majority of patients (Roosendaal et al., 2010).

The major clinical subtypes of MS are relapsing-remitting (RR) and secondary progressive (SP) MS. RR-MS represents the initial inflammatory phase of approximately 85–90% of all cases. SP-MS usually develops later in the disease course and is associated with axon degeneration (Lublin and Reingold, 1996). A less common progressive subtype is primary progressive (PP) MS. PP-MS affects approximately 10% of all cases and manifests with no relapses but a steady decline in function from disease onset (Andersson et al., 1999).

For treatment of MS, during the past decades, attention was paid to the modulation of immune responses for prevention of axon demyelination. Immunomodulatory and anti-inflammatory agents can efficiently slow down RR-MS (and SP-MS to a certain extent) progression by reducing the frequency of relapses (Fischer et al., 2000; Barak and Achiron, 2002; Goodin et al., 2002). However, they do not improve disease outcome after degeneration occurs, and therefore are insufficient to treat chronic neurological disability in patients with progressive disease (Molyneux et al., 2000; Compston and Coles, 2002; Coles et al., 2006).

It is now known, that treatment of MS requires not only prevention, but also repair of the injury. Good candidates for the repair of MS lesions are the neural stem cells (NSCs), cells that retain their multipotential capacity in the adult and senescent brain (Van den Berge et al., 2010). In this review we will highlight the importance that regenerative capacity of endogenous NSCs has in future therapeutic options for MS.

## Neural stem cells in the adult mammalian brain

NSCs are multipotent cells that have the ability to self-renew and differentiate into neurons or glial cells (Gage, 2000). Their production is mainly taking place at the neural tube of the developing brain (Wilson and Stice, 2006). NSCs persist in the adult brain, albeit in much lower densities, being also present in the brains of elderly and patients with a neurodegenerative disorder (Leonard et al., 2009; Van den Berge et al., 2010, 2011). In the adult brain, generation of NSCs is restricted in two highly specialized tissue niches: the subventricular zone (SVZ) of the lateral ventricles and the subgranular zone (SGZ) of the hippocampus (Gage et al., 1998; Quiñones-Hinojosa et al., 2007). Newborn NSCs enter an active phase of proliferation and/or differentiation once they receive stimulatory signals, such as growth factors produced by the surrounding cells (Gage et al., 1998; Sun et al., 2010). Differentiation of NSCs can result in the production of new neurons with neurogenesis, or glial cells with gliogenesis.

### Neurogenesis

Animal studies have shown that NSCs generated at the adult hippocampal SGZ form new neurons which integrate in the dentate gyrus (Cameron and Gould, 1994) whereas, NSCs generated at the adult SVZ, form new interneurons which integrate in the olfactory bulb (OB) (Carleton et al., 2003). The adult human SVZ is characterized by a dense ribbon of glial fibrillary acidic protein (GFAP) positive astrocytes that lines the lateral wall of the lateral ventricles. This astrocyte ribbon is highly proliferative and clearly separated from the ependyma by a hypocellular gap layer (Nader Sanai, 2004).

New SVZ-derived neuroblasts which were differentiated from NSCs migrate to the OB through the rostral migratory stream (RMS) in rodents and non-human primates (Craig et al., 1999; Pencea et al., 2001a). In this stream, migrating neuroblasts build elongated chains that are tangentially oriented to the OB, through glial tubes formed by astrocytes (Lois et al., 1996). In humans however, migration of neuroblasts is elusive; studies in the early postnatal brain described a primary corridor connecting the infant SVZ to the OB, and a branching stream that reaches the ventromedial prefrontal cortex (Sanai et al., 2011); studies in the adult human brain indicated the presence of the primary corridor only and described migration of a modest number of neuroblasts (Van Strien et al., 2011).

In MS, neurogenesis possibly occurs at the SVZ leading to generation of new immature neurons in a subgroup of chronic subcortical lesions (Chang et al., 2008). However, neurogenesis in the MS brain is reduced compared to healthy adult brain, leading to lower neuronal supply to the OB which might explain the often reported olfactory deficits in patients (Tepavčević et al., 2011).

### Gliogenesis

In the developing brain gliogenesis follows neurogenesis but persists long after neurogenesis has ceased (Jacobson, 1991). Differentiation of glial progenitor cells results in the formation of astrocytes or oligodendrocytes (Lee et al., 2000). Oligodendrogenesis in adulthood is restricted in and around the SVZ and involves the formation of an intermediate progenitor phenotype, the oligodendrocyte progenitor cell (OPC). OPCs are self-renewing cells that can reside in multiple areas of the adult healthy brain parenchyma until they get activated and differentiated (Menn et al., 2006). Differentiation of OPCs involves expression of specific markers, changes in cellular morphology and extension of endfeet toward the axons for myelination (Compston et al., 1997; Blakemore and Keirstead, 1999; Young et al., 2013).

In MS, loss of axon myelin is often followed by the remyelination of nude axons with new myelin sheaths. Remyelination is a naturally regulated process orchestrated by mature oligodendrocytes (Blakemore and Keirstead, 1999). This process is activated in response to acute demyelination and often leads to formation of shadow plaques, areas of complete repair activity, in otherwise intact white or gray matter. Remyelination activity is high in acute (average 80.7% of all lesions) and persists in chronic progressive MS (average 60% of all lesions). In the WM, remyelination occurs mainly in early inflammatory (Goldschmidt et al., 2009) and chronic active lesions (Patani et al., 2007), the latter being lesions surrounded by a sharp border of activated microglia/macrophages. For remyelination in MS, resident OPCs are recruited and differentiated into mature oligodendrocytes (Gensert and Goldman, 1997; Nait-Oumesmar et al., 1999); moreover, new oligodendrocyte lineage cells are produced by a two to three-fold activation of the adult SVZ in MS (Nait-Oumesmar et al., 2007) and EAE (Picard-Riera et al., 2002; Tepavčević et al., 2011).

## Remyelination by parenchymal OPCs in MS

Remyelination, although activated in MS, is insufficient to repair severe and long-lasting demyelination events like the ones occurring in the progressive phases of the disease. The failure of remyelination to sufficiently restore chronic damage is not caused by the lesional depletion of OPCs (Wolswijk, 1998); instead, changes that can be induced at any of the four phases consisting the remyelination process, may reduce capacity of endogenous repair in various ways that are discussed below (Franklin, 2002).

### Proliferation of OPCs

In the adult demyelinating brain activated astrocytes and microglia secrete mitogens that induce OPC proliferation, such as the platelet-derived growth factor receptor-2A (PDGF-2A) and fibroblast growth factor-2 (FGF-2) (Franklin and ffrench-Constant, 2008; Clemente et al., 2013). The response of OPCs to mitogens is regulated by the cell cycle regulatory protein p27-Kip1 (Crockett et al., 2005) and the cyclin-dependent kinase 2 (Caillava and Baron-Van Evercooren, 2012). In MS changes in the mitogenic environment induced by alterations in the levels of secreted factors might inhibit proliferation of OPCs (Franklin, 2002). Modulation of growth factor levels such as the FGF-2, was shown to enhance OPC proliferative activity in several *in vitro* models (Armstrong et al., 2002; Dziembowska et al., 2005).

### Migration of OPCs

Animal studies showed that inflammation and demyelination promote migration of OPCs to the lesions (Nait-Oumesmar et al., 1999; Piao et al., 2013). OPCs migrate to early inflammatory and chronic active MS lesions possibly being attracted by FGF-2 expressed by infiltrating macrophages and microglia-derived macrophages (Clemente et al., 2011). In chronic inactive lesions however, formation of the characteristic glial scar composed of hypertrophic astrocytes, limits access of OPCs to the lesion center (Franklin and ffrench-Constant, 2008). Manipulation of chemotactic pathways such as the C-X-C chemokine receptor type 4, pharmacogenetic targets such as the FGF-2 and Anosmin-1, or guidance cues such as semaphorin-3A and 3F, might promote repopulation of MS lesions by OPCs, as it was shown in experimental *in vivo* or *in vitro* models for MS and in human MS brain tissue (Williams et al., 2007; Carbajal et al., 2010; Clemente et al., 2011).

### Differentiation of OPCs

Demyelination influences the capacity of OPCs to differentiate by forming a “dysregulated” signaling environment. In chronic MS lesions, OPCs often acquire an immature phenotype which is not permissive for axon remyelination (Wolswijk, 2000). Events that inhibit OPC differentiation are the (1) Excessive accumulation of myelin fragments (Kotter et al., 2006), (2) Deposition of hyaluronan (Sloane et al., 2010), (3) Dysregulation of signaling pathways controlling cell fate, such as the Wnt/β–catenin (Feigenson et al., 2011) and the Notch–Jagged pathway (John et al., 2002; Nakahara et al., 2009) and (4) Changes in levels of growth factors or bone morphogenic proteins (Franklin, 2002; Cheng et al., 2007). Pharmacological manipulation of therapeutic targets such as leucine rich repeat and Ig domain containing 1 (Jepson et al., 2012), retinoic X receptors (Huang et al., 2011), phosphodiesterase-7 (Medina-Rodríguez et al., 2013) and cyclin-dependent kinase 5 (Cdk5) (Luo et al., 2014) or signaling pathways such as the Fyn-Rho-ROCK and protein kinase C (PKC) (Baer et al., 2009) and the Notch/Jagged1 (Blanchard et al., 2013), can be advantageous for the repair of MS lesions.

### Function of mature oligodendrocytes

In the brain of MS patients, mature oligodendrocytes may undergo demyelination thereby losing capacity to myelinate (Wolswijk, 2000). Moreover, demyelinated axons may lose receptivity to myelination by oligodendrocytes due to re-expression of negative regulators, such as the polysialylated neural cell-adhesion molecule (PSA-NCAM) (Charles et al., 2002). These two or other events such as the reported reduction in astrocytic expression of neuregulins (Viehover et al., 2001) or the increased expression of insulin-like growth factor (IGF) binding proteins by oligodendrocytes (Wilczak et al., 2008), might prevent restoration of axon conduction properties in MS lesions. Pharmacological interventions to reverse those events might prove efficient in promoting repair of MS lesions (Lundgaard et al., 2013).

## Regeneration by new cells in MS

### Targeting oligodendrocyte regeneration

Since sufficient levels of remyelination can repair damaged axons, stimulation of endogenous activity might be beneficial for repair of MS lesions. Besides activation of parenchymal OPCs, generation of new oligodendrocytes for the increase of brain remyelination activity appears a promising perspective for future therapies. This can be achieved with engraftment of transplanted cells or with stimulation of SVZ-derived NSCs by transplanted cells or growth factors to induce differentiation into OPCs.

#### Stimulation of endogenous NSCs with cell transplantation

Transplanted stem cells can boost remyelination by cell replacement or bystander neuroprotection and/or immunomodulation (Pluchino and Martino, 2008). Bystander effects induced by transplanted cells were shown to promote migration, differentiation and/or survival of endogenous NSCs thereby enhancing self-repair. The ideal cell types for transplantation in animal models of demyelination are the NSCs and mesenchymal stem cells (MSCs), while two other possible cell sources are the embryonic and induced pluripotent stem cells (iPSCs). The route of administration is an important factor determining the effect type and magnitude that cell transplantation induces. Mostly preferred routes for the delivery of MSCs or NSCs are the intravenous and intrathecal injections (Gianvito Martino, 2010). Administration of autologous MSCs in patients with progressive MS was shown to improve clinical outcome based on magnetic resonance imaging (MRI) examination while reported adverse effects were not significant (Laroni et al., 2013).

#### Stimulation of endogenous NSCs with growth factors

The new thrust in the treatment of MS is grounded on the discovery that NSCs persist within the adult healthy brain. The human SVZ is located at the lateral wall of the lateral ventricles, thereby providing an accessible route for the intracerebroventricular delivery of growth factors to activate NSC proliferation/differentiation. Newborn cells may theoretically replace the old damaged ones and promote repair in areas with poor regeneration. Intracerebroventricular application of growth factors have revealed multiple beneficial effects in EAE (Tafreshi, 2006).

**Figure 1:**
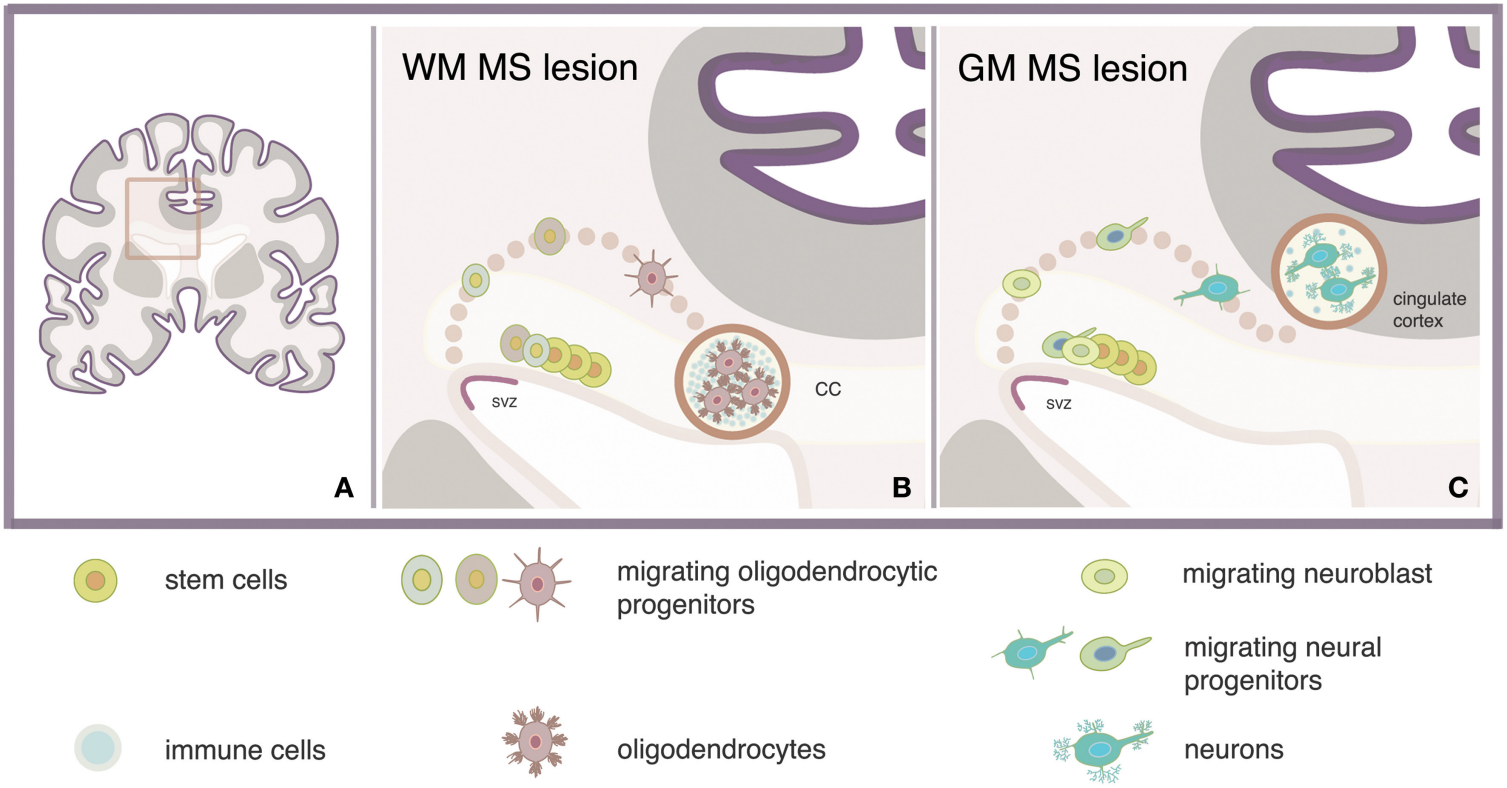
**Model strategy for regeneration of MS lesions via activation of adult SVZ-derived NSCs**. Stimulation of neural stem cells (NSCs) via intracerebroventricular administration of, e.g., growth factors may lead to the regeneration of newly formed lesions which are located in white matter (WM) or gray matter (GM) areas nearby the subventricular zone (SVZ) of the multiple sclerosis (MS) brain **(A)** such as the adjacent corpus callosum (CC) **(B)** and the cingulate gyrus **(C)**.

The regeneration of MS lesions with new oligodendrocytes depends on the stage and location of the lesion. Although the role of inflammation in remyelination has not yet been clarified, our speculation is that repair of small newly-formed lesions might be advantageous over the repair of chronic lesions, in terms of reparative efficiency and energy consumption. Periventricular lesions are often present in MS and analyses of such lesions showed enhanced number of pro-migratory adhesion molecule PSA-NCAM+ progenitors expressing Sox9, Sox10, and/or Olig2 markers of glial fate. Concomitant enhancement of the PSA-NCAM+ progenitor population at the proximal SVZ suggested increased oligodendrogenesis (Nait-Oumesmar et al., 2007). These pieces of evidence indicate that regeneration of oligodendrocytes might be efficient in repairing lesions located near the SVZ, such as the corpus callosum (Figure 1). In accordance, recent evidence showed a four-fold increase in the number of NSC-derived oligodendrocytes expressing the PDGF receptor α and Olig2 markers of oligodendrocytes, in a single demyelinating lesion within the corpus callosum (Menn et al., 2006).

Interventions to modulate growth factor signaling for promoting oligodendrocyte replacement and remyelination are nowadays the focus of intense study. Both *in vivo* and *in vitro* experiments revealed various factors which act on OPCs derived by the adult SVZ, and influence proliferation, migration or differentiation properties (Table 1). An advanced candidate for MS is IGF-1, a factor previously shown to delay EAE onset and improve clinical outcome (Zhang et al., 2011). In a pilot trial, seven MS patients were treated with subcutaneous administration of recombinant (rh) IGF-1 over a 6-month period of time. The peripheral administration of rhIGF-1 had no significant adverse effects. However, MRI examination of patients after treatment showed no significant clinical improvement. Explanations for the absence of positive effects on clinical outcome were the small sample size and the limited penetration of rhIGF-1 across the BBB (Frank et al., 2002). Since surgically invasive procedures are not applicable in human trials, intranasal administration of growth factors might represent an interesting alternative approach for successful delivery inside the CNS (Hanson and Frey, 2008).

**Table 1:** **Factors and secreted molecules activating the adult SVZ-derived NSCs, in animal models of demyelination**.

**Factor**	**Model**	**Process**	**Function**	**References**
HB-EGF	LPC demyelinated mouse	O	Recruitment	Cantarella et al., 2008
FGF-2	Cell culture	O	Recruitment	Clemente et al., 2011
CNTF	LPC demyelinated rodent	O	Recruitment	Vernerey et al., 2013
NGF	EAE rat	O	Differentiation	Aloe and Micera, 1998
IGF-1	Cell culture	N	Differentiation	Brooker et al., 2000
PEDF	Transgenic mouse	O	Fate commitment	Sohn et al., 2012
PDGF	Transgenic mouse	O	Proliferation	Jackson et al., 2006
VEGF	Unlesioned rat	N	Proliferation	Jin et al., 2002
BDNF	Unlesioned rat	N	Proliferation	Pencea et al., 2001b
**MOLECULE**				
Reelin	LPC demyelinated mouse	N	Recruitment	Courtès et al., 2011
Netrin 1	LPC demyelinated mouse	O	Recruitment	Cayre et al., 2013
Chordin	LPC demyelinated mouse	O	Recruitment	Jablonska et al., 2010
Noggin	Cuprizone demyelinated mouse	O	Proliferation	Cate et al., 2010
			Fate commitment	

*LPC, Lysolecithin; O, Oligodendrogenesis; N, Neurogenesis; EAE, experimental autoimmune encephalomyelitis; HB-EGF, Heparin-binding epidermal growth factor; FGF-2, Fibroblast growth factor 2, CNTF; Ciliary neurotrophic factor; NGF, Nerve growth factor; IGF-1, Insulin-like growth factor 1; PEDF, Pigment epithelium-derived factor; PDGF, Platelet-derived growth factor; VEGF, Vascular endothelial growth factor; BDNF, Brain-derived neurotrophic factor*.

### Targeting neuronal regeneration

The confirmed existence of OPCs in the adult brain as well as the increasing understanding of the pathways regulating endogenous remyelination, are the basic reasons why regeneration of oligodendrocytes is a more well investigated approach compared to regeneration of neurons. Replacement of damaged neurons however, is anticipated to offer intriguing possibilities for the rehabilitation of cognitive disturbances in patients with progressive MS. New neurons may repopulate focal sites of degeneration located near the SVZ, such as the OB or the cingulate gyrus, to induce repair, contributing to the re-growth of nerves that have been lost (Figure 1).

Challenges associated with that strategy are linked to the type, density and ability of new neurons to integrate into defined circuits. Acquisition of specific neuronal subtypes is a demanding step for neuronal regeneration of MS lesions since recent evidence indicated heterogeneity and reduced neuropotency in the population of adult SVZ-derived NSCs (Shen et al., 2006). Moreover, the ability of new neurons to functionally mature at the sites of damage is still debated because the sites of lesions are normally non-neurogenic (Obernier et al., 2014). For the expansion of new neurons, and promotion of maturation, specific growth factors can be administered at the SVZ (Table 1). The possibility that new neurons are poorly myelinated due to lack of oligodendrocytes has to be studied. Importantly, the extraordinary proliferation or direction of SVZ-derived NSCs toward neuronal phenotypes other than the ones they were intrinsically committed for might induce unwanted effects linked to tumorigenesis. Monitoring and quantification of NSC activation is required and can be conducted with non-invasive techniques, such as the positron emission tomography (Rueger et al., 2010).

## Conclusion

Stimulation of endogenous NSCs with growth factors is an interesting approach for treatment of MS and requires more research in order to reveal its entire therapeutic potential. An important question that needs to be addressed is if this approach can repair all or subtypes of MS lesions depending on whether the damage is focal or diffuse. Notably, MS is a complex disease showing activity even in late progressive phases. Therefore, even if regeneration with NSCs proves efficient to revert damage in the CNS of MS patients that today is considered to be irreversible, a combination with disease modifying agents might be needed to halt MS progression.

### Conflict of interest statement

The authors declare that the research was conducted in the absence of any commercial or financial relationships that could be construed as a potential conflict of interest.
